# miR-204 suppresses the development and progression of human glioblastoma by targeting ATF2

**DOI:** 10.18632/oncotarget.11732

**Published:** 2016-08-31

**Authors:** Shiwei Song, Abul Fajol, Xiankun Tu, Baogang Ren, Songsheng Shi

**Affiliations:** ^1^ Department of Neurosurgery, Fujian Medical University Affiliated Union Hospital, Fujian Neurosurgical Institute, Fuzhou, 350001, China; ^2^ Department of Clinical Sciences, CRC, Lund University, Malmö, 20502, Sweden

**Keywords:** miR-204, GBM, suppressor

## Abstract

In human cancers, miRNAs are important regulators of multiple cellular processes, and aberrant miRNA expression has been observed, and their alterations contribute to multiple cancer development and progression. Till now, little has been known about the role of miR-204 in human glioblastoma (GBM). In the present study, we used in-vitro assays to investigate the mechanisms of miR-204 in GBM cell lines and 60 cases of GBM tissues. Here, we found that miR-204 expression is downregulated in both GBM cell lines A172, U87 and U251 cells and GBM tissues as compared with NHA cells and normal tissues (all p<0.001). In addition, the ectopic expression of miR-204 suppressed A172 and U87 cell proliferation, migration and invasion. Meanwhile, miR-204 over-expression extremely inhibited the protein expression of ATF2. Notably, the enforced expression of ATF2 in A172 and U87 cells with the over-expression of miR-204 attenuated the inhibitory effects of miR-204 on proliferation, migration and invasion. In conclusion, our findings suggest that miR-204 suppressed cell proliferation, migration and invasion through inhibition of ATF2, thus, miR-204 may function as a useful drug target in the treatment and diagnosis of GBM.

## INTRODUCTION

Glioblastoma multiforme (GBM) is a grade IV astrocytoma and the most lethal primary brain tumor [1]. Astrocytomas are graded based on nuclear atypia, mitosis, vascular endothelial proliferation and necrosis, which define the diagnosis criteria of GBM [2]. GBM is featured with a large degree of tumor heterogeneity and easy invasion into surrounding tissues [3, 4]. The median survival time for GBM is only 14.6 months with a 2-year survival rate of 26%, although they have been remarkably improved [5]. Although some potential drug targets have been discovered, including transforming growth factor-β, epidermal growth factor receptor, phosphatase and tensin homolog etc, the lethality of GBM is not significantly changed due to the efforts [6, 7]. Thus, there is still much urgency for new and effective biomarkers to help find more therapeutic targeted drugs.

The miRNAs are reported as a cluster of small, and non-coding RNAs, and have the capacity of regulating the expression of some genes at both the transcriptional and translational levels [8–11]. The miRNAs are involved in various cellular processes of cancer development, including cell proliferation, differentiation, migration and invasion [12–15]. Emerging evidence has identified that the deregulation of miRNAs is related to initiation of various cancers, such as bladder cancer, gastric cancer, lung cancer, and breast cancer [16, 17]. Accumulating studies showed that the deregulated expression of miR-204 was observed in various cancers. For example, miR-204 was reported to be significantly upregulated in most pancreatic cancer [18]. Recently, miR-204 function as a tumor suppressive miRNA and miR-204 expression level is down-regulated in various human malignancies: endometrial cancer [19], prostate cancer [20], medulloblastomas [21], non-small cell lung carcinoma [22, 23]. However, the expression and mechanism of miR-204 in bladder cancer remain unclear.

In the present study, we investigated the potential role of miR-204 in GBM cancer progression using in-vitro assays like RT-PCR and Western blot. We showed that miR-204 is downregulated in clinically obtained human GBM tissues. Moreover, we explored that miR-204 plays a crucial role in cell proliferation, migration and migration by directly targeting ATF2 in GBM cells. Our data suggest a novel molecular mechanism of the tumor suppressor activity of miR-204. Re-expressing miR-204 and/or interfering with ATF2 function might be a promising therapy strategy.

## RESULTS

### miR-204 expression is reduced in GBM cell lines and tissues

**Figure 1:**
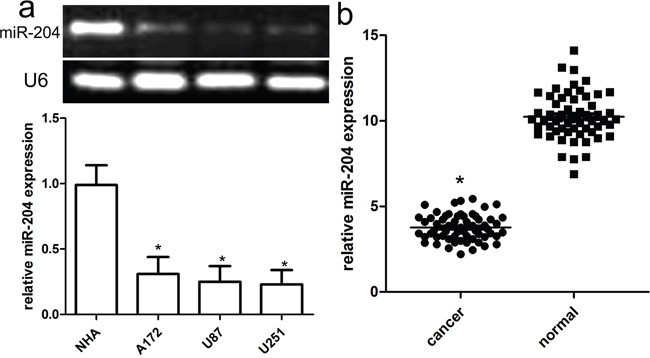
Reduced miR-204 expression in GBM cell lines and tissues **a.** Relative miR-204 expression in GBM cell lines (A172, U87, and U251) and a normal human brain cell line NHA. **b.** Relative miR-204 expression in 60 pairs of GBM tissues and adjacent normal counterpart tissues was detected using real-time RT-PCR. *p<0.001, vs NHA or normal tissues.

miR-204 expression was detected by qRT-PCR in GBM cell lines (A172, U87, and U251) and a normal human brain cell NHA. All GBM cancer cell lines tested had lower miR-204 levels than did the NHA cells (Figure 1a). Of 60 GBM samples, miR-204 was obviously down-regulated compared with the adjacent normal tissues (Figure 1b).

### miR-204 inhibits GBM cancer cell proliferation, migration and invasion

**Figure 2:**
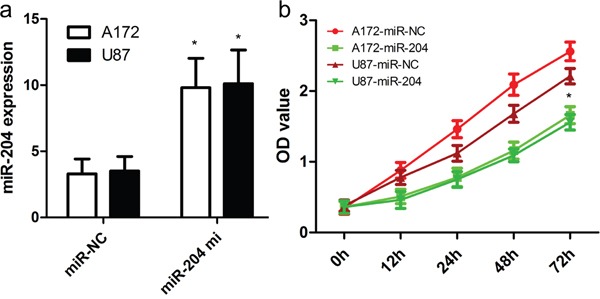
miR-204 inhibits GBM cell proliferation **a.** Relative miR-204 expression in A172 and U87 cells was measured after the cells were transfected with miR-204 mimics or scramble control miRNA using real-time RT-PCR. **b.** Cell proliferation was measured using a CCK-8 assay. A172 and U87 cells were transfected with miR-204 mimics or scramble control miRNA. *p<0.001, vs control.

**Figure 3:**
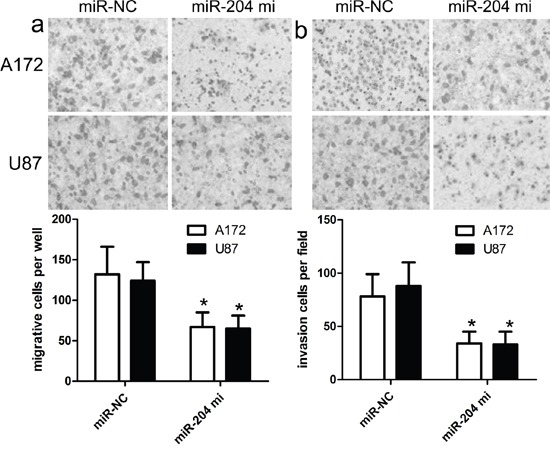
miR-204 inhibits GBM cell migration and invasion **a.** The migration capacity of A172 and U87 cells was measured by transwell migration assay after transfecting the cells with miR-204 mimics or scramble control miRNA for 48 h. Overexpresion of miR-204 inhibited the invasion of A172 cells. The relative ratio of invasive cells per field is shown. **b.** The invasive capacity of A172 and U87 cells was assessed by transwell invasion assay after transfecting the cells with miR-204 mimics or scramble control miRNA for 48 h. Overexpresion of miR-204 inhibited the invasion of A172 and U87 cells. The relative ratio of invasive cells per field is shown. *p<0.001, vs control.

To figure out the role of miR-204 in GBM cell proliferation, we generated miR-204-overexpressing A172 and U87 cells by transiently transfecting cells with miR-204 mimics. miR-204 expression was confirmed by real time RT-PCR (Figure 2a). Our findings showed that miR-204 overexpression resulted in significantly reduced cell proliferation in both A172 and U87 cells (Figure 2b). To figure out the role of miR-204 in GBM cell migration and invasion, tranwell migration and invasion assay was performed to assess the effects of miR-204 on the migration and invasion capacity of A172 and U87 cells. The tranwell assay revealed that miR-204 overexpression repressed the migration and invasion capacity of A172 and U87 cells compared with that of cells transfected with the miR-NC control (Figure 3a, 3b).

### ATF2 is identified as a target of miR-204

**Figure 4:**
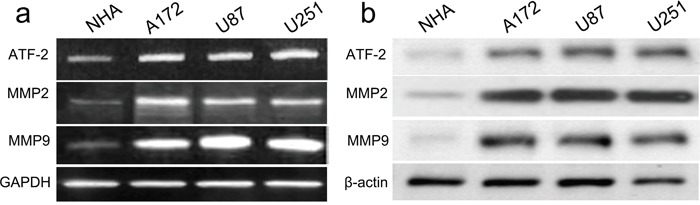
ATF2, MMP2 and MMP9 were up-regulated in GBM cell lines and NHA cells The mRNA and protein level of ATF2, MMP2 and MMP9 was measured in GBM cell lines using western blot. β-actin and GAPDH was used as an internal loading control. The mRNA and protein expression level was calculated using Image J Pro software. Each bar represents the mean ± SD of three independent experiments; *p<0.001, compared with NHA.

**Figure 5:**
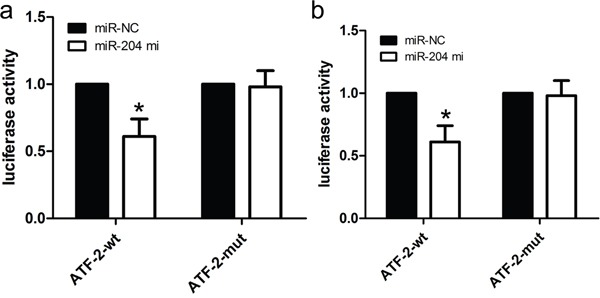
ATF2 is a candidate target of miR-204 **a.** The miR-204 mimics inhibited the luciferase activity controlled by wild-type ATF2-3'-UTR, but did not affect the luciferase activity controlled by mutant ATF2-3'-UTR in A172 cells. **b.** The miR-204 mimics inhibited the luciferase activity controlled by wild-type ATF2-3'-UTR, but did not affect the luciferase activity controlled by mutant ATF2-3'-UTR in U87 cells. *p<0.001, vs. miR-NC control.

A putative miR-204 binding site was detected at the end of the ATF2 3'UTR according to previous reports. Firstly, ATF2 expression was detected by real time PCR and western blot in GBM cell lines (A172, U87, and U251) and NHA cells. All GBM cell lines had higher mRNA and protein expression of ATF2 than did the NHA cells (Figure 4a, 4b). Accompanied by identification of ATF2, the expression of MMP2 and MMP9 were also detected, whose expression profile was consistent with ATF2. In addition, in comparison with ATF2 mutation-type 3'UTR, the luciferase reporter activity was decreased by approximately 40% in A172 cells or 45% in U87 cells containing the ATF2 wild-type 3'UTR fragment (Figure 5a, 5b). Besides, we made a correlation analysis to elucidate the negative association of miR-204 and ATF2 in A172, U87, and U251 cells.

### Enforced ATF2 attenuates the inhibitory effects of miR-204

**Figure 6:**
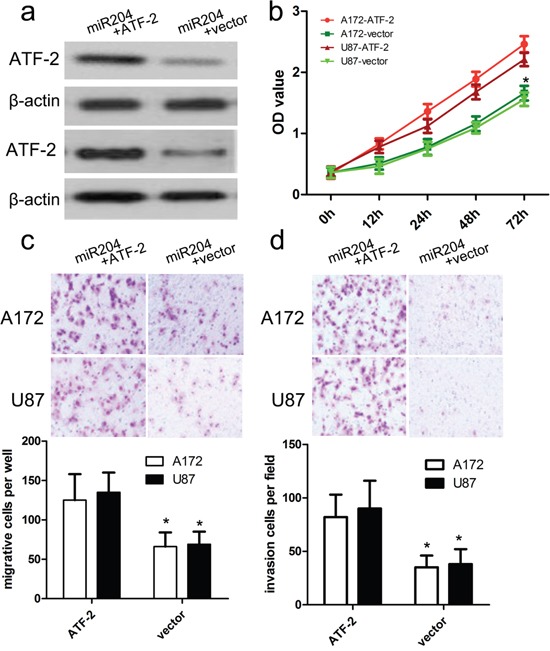
ATF2 overexpression reversed the inhibitory effect of miR-204 on GBM cell proliferation, migration and invasion **a.** Western blot analysis showed that transfecting the cells with ATF2 plasmids could up-regulate ATF2 expression. The expression of ATF2 was normalized to that of β-actin. **b.** The proliferation capacity of miR-204-overexpressing A172 and U87 cells was partially improved when cells were transfected with ATF2 plasmids in comparison with miR-NC. **c, d.** The migration and invasion of miR-204-overexpressing A172 and U87 cells were effectively improved when cells were transfected with ATF2 plasmids. *p<0.001, vs. vector.

To further elucidate the association between miR-204 and ATF2, we conducted a transfection pcDNA3.1(+)-ATF2 into miR-204-overexpressing A172 and U87 cells to enhance the overexpression of ATF2 protein (Figure 6a). The CCK-8 proliferation assay showed that ATF2 overexpression enforced the proliferation potentials of A172 and U87cells (Figure 6b). What is more, our transwell assay also showed that ATF2 overexpression in miR-204-overexpressing A172 and U87 cells enhanced the migration and invasion capacity of A172 and U87 cells in comparison with vector control (Figure 6c, 6d).

## DISCUSSION

In this study, we examined both the regulation of the ATF2 pathway by miR-204 in GBM, as well as its functional significance. The miR-204 has been commonly deregulated in various cancers. For example, miR-204 was reported to be significantly upregulated in most pancreatic cancer. Recently, miR-204 function as a tumor suppressive miRNA, and miR-204 expression level is down-regulated in endometrial cancer, prostate cancer, medulloblastomas, non-small cell lung carcinoma. However, little is known of its expression and potential function in GBM. Here, we reported down-regulation of miR-204 and demonstrate its role as a tumor suppressor in GBM. These finding also indicated that miR-204 is implicated in the development of GBM.

Next, we further demonstrated that miR-204 inhibited ATF2 expression by targeting its 3'UTR, indicating that ATF2 is indeed a direct target of miR-204. Subsequently, we carried out western blot to testify the overexpression of miR-204 could inhibit ATF2 protein expression. Some reports also identified ATF2 is a key signal molecule involved in cancer progression and development [24, 25]. Here, we found that ATF2 overexpression reverses the inhibitory effects of over-expressed miR-204 on GBM cancer cell proliferation, migration and invasion. Thus, the present data demonstrates that miR-204 regulates ATF2 expression, and thus over-expressed miR-204 functions as a tumor suppressor in GBM progression.

Besides, we demonstrated that MMP2 and MMP9 were highly expressed in GBM cells, accompanied by up-regulated ATF2 expression. According to reports, MMPs act as a kind of calcium-dependent zinc-containing endopeptidases, and plays a crucial role in the remodeling of organs and degradation of the matrix. Increase in matrix metalloproteinases in different tumors and their correlation with tumor invasiveness have been documented. Meanwhile, ATF2 also has been reported to regulate the tumor behaviors. Thus, it should be inferred that miR-204 affected proliferation, migration and invasion directly or indirectly via inhibition of ATF2 to affect the expression of MMP2 and MMP9.

In conclusion, our work demonstrates that miR-204 is frequently downregulated in patients with GBM, and can be recommended as a potential tumor-suppressing miRNA to inhibit GBM development and progression. ATF2 regulated by miR-204 might also play an important role in the regulation of malignant behavior of GBM. Therefore our study provides a novel and promising therapeutic target for GBM treatment.

## MATERIALS AND METHODS

### Ethics statement

All of the patients provided written informed consent. This study was approved by the Ethics Committee of Fujian Medical University Affiliated Union Hospital, and complied with the Declaration of Helsinki.

### GBM cell lines and tissues

Human glioblastoma cell lines A172, U251 and U87 were purchased from Cell Bank of the Chinese Academy of Sciences (Shanghai, People's Republic of China). All these cells were cultured in Dulbecco's Modified Eagle's Medium containing 10% fetal bovine serum supplemented with streptomycin (100 μg/mL) and penicillin (100 U/mL). Primary GBM tissues and adjacent normal GBM tissues (more than 3 cm away from the tumor) were collected during surgery in Jinan Central Hospital. None of the patients had received chemotherapy before surgical resection.

### RNA isolation and quantitative RT-PCR

Total RNA was isolated from tissues and cell lines using the miRNeasy Mini Kit (Qiagen). The miRNA Q-PCR Detection Kit (GeneCopoeia) was used for quantification of miRNA levels according to the manufacturer's protocol. The protocol was conducted for 35 cycles at 95°C for 3 min, 95°C for 12 s, and 58°C for 30 s. The PCR amplification for the quantification of the miR-204 and U6 was performed using TaqMan miRNA Reverse Transcription Kit (Applied Biosystems, Foster City, CA, USA) and TaqMan Human MiRNA Assay Kit (Applied Biosystems, Foster City, CA, USA). The following primers were used:

miR-204 mimics, forward: 5′-GCGGCGCAAA GAATTCTCCT-3′ and reverse: 5′-GTGCAGGGT CCGAGGT-3′;

U6 forward: 5'-CTCGCTTCGGCAGCACA- 3', and reverse: 5'-AACG CTTCACGAATTTGC GT-3';

ATF2 forward 5'-GCAACGACCGTAATCG CATC-3' and ATF2 reverse 5'-CCATTGCCGGC TAGGGTTTA-3';

GAPDH forward 5'-TTGATGGCAACAAT CTCCAC-3' and GAPDH reverse 5'-CGTCCCG TAGACAAAATGGT-3'.

The reaction conditions were as follows: 95°C for 30 seconds, followed by 48 cycles of 95°C for 5 seconds, 60°C for 10 seconds and 72°C for 30 seconds. Relative miR-204 expressions were calculated with normalization to U6, and GAPDH was used to as internal controls. The relative expression was calculated by the 2-ΔΔT method. And the relative expression was calculated by the ratio of U6 or GAPDH.

### Western blot analysis

Total proteins were isolated from cells or tissues, and protein concentrations were detected using the BCA Assay Kit (Thermo Scientific). Protein samples were separated by 10% SDS-PAGE and transferred onto PVDF membranes (Millipore). The membranes were blocked with 5% non-fat milk for two hours and then incubated overnight at 4°C with the primary antibody, including ATF2 (Cell Signaling Technology, 1:1000), MMP2 (Cell Signaling Technology, 1:1000), MMP9 (Cell Signaling Technology, 1:1000) and β-actin (Santa Cruz Biotechnology, 1:1000). Signals were detected using the enhanced chemiluminescence (ECL) luminol reagent (PerkinElmer Inc.). β-actin was used as a loading control.

### miRNA mimic and inhibitor transfection

Functional consequences of aberrant miR-204 expression were studied by transiently transfecting miR-204 mimic (Life technologies, Shanghai, China), and corresponding negative controls miR-NC (GenePharma, Shanghai, China) into cells. A172 and U87 cells were seeded on a 24-well plate at 10,000 cells per well and transfected 24 hours later with an miRNA mimics at a final concentration of 10 nm using Lipofectamine 2000 (Invitrogen, Carlsbad, CA, USA) according to the manufacturer's instructions. At 48 hours after transfection, A172 and U87 cells were harvested for western blot or qRT-PCR analyses.

### Transfection of plasmids

A172 and U87 cells were transfected using Lipofectamine 2000 transfection reagent (Invitrogen, Carlsbad, CA). ATF2 cDNA without its 3'UTR (4,017 bp) was inserted into pcDNA3.1(+) to generate the recombinant vector pcDNA3.1(+)-ATF2. All constructs were verified for sequence correctness by direct sequencing (Beijing Aodingsheng Corp., Beijing China).

### Cell proliferation

For the cell proliferation assay, A172 and U87 cells were plated into 96-well plates and cultured for 0, 1, 2 or 3 days, after which CCK-8 (10 μL) was added to each well. A172 and U87 cells were then incubated for an additional 2 h, and the absorbance was then detected at a wavelength of 450 nm.

### Transwell assays

A172 and U87 cells were plated in the upper chambers of Matrigel-coated wells (1:5 dilution in serum-free medium), and medium supplemented with 10% serum was added to the lower chamber. After culturing for 24 h, A172 and U87 cells remaining on the upper surface of the membranes were removed, and the cells on the lower surface of the chamber were then stained with 0.1% crystal violet (Sigma) and counted.

### Luciferase reporter assay

The 3′-UTR sequence of ATF2 was amplified from normal human genomic DNA and subcloned into the pmirGLO luciferase reporter vector (Promega). Cells (3.5 × 10^4^) were seeded in triplicate in 24-well plates and cotransfected with wild-type (wt) or mutant (mut) 3′-UTR vectors and miR-204 mimics using Lipofectamine 2000. After 48 h, A172 and U87 cells were assayed for luciferase activity using the Dual-Luciferase Reporter Assay System (Promega) by following the manufacturer's instructions. The firefly luciferase activities were normalized to Renilla luciferase activity. The firefly luciferase activity of the cells that were transfected with miR-204 mimics or miR-NC is represented as the percentage of activity relative to that of cells that were transfected with negative controls. All experiments were performed in triplicate.

### Statistical analysis

All the data were shown as the mean ± SD, and difference were determined by two-tailed Student's t-test of SPSS. For comparison in multiple groups, one-way ANOVA was applied. The LSD and SNK methods were used when homogeneity of variance, while Tamhane's T2 or Dunnett's T3 method was considered when heteroskedasticity. P < 0.05 was considered as statistically significant, and P < 0.01 was regarded as strong significant.
